# Ambient air pollution and emergency department visits in Toronto, Canada

**DOI:** 10.1007/s11356-021-12519-3

**Published:** 2021-02-06

**Authors:** Mieczysław Szyszkowicz, Nicholas de Angelis

**Affiliations:** 1 Environmental Health Science and Research Bureau, Health Canada, Ottawa, Canada; 2 Biomedical Program, Department of Mechanical and Aerospace Engineering, Carleton University, Ottawa, Canada

**Keywords:** Ambient air pollution, AQHI, Case, Concentration, Counts, Emergency department, Relative risk

## Abstract

To investigate the acute impact of various air pollutants on various disease groups in the urban area of the city of Toronto, Canada. Statistical models were developed to estimate the relative risk of an emergency department visit associated with ambient air pollution concentration levels. These models were generated for 8 air pollutants (lagged from 0 to 14 days) and for 18 strata (based on sex, age group, and season). Twelve disease groups extracted from the International Classification of Diseases 10th Revision (ICD-10) were used as health classifications in the models. The qualitative results were collected in matrices composed of 18 rows (strata) and 15 columns (lags) for each air pollutant and the 12 health classifications. The matrix cells were assigned a value of 1 if the association was positively statistically significant; otherwise, they were assigned to a value of 0. The constructed matrices were totalized separately for each air pollutant. The resulting matrices show qualitative associations for grouped diseases, air pollutants, and their corresponding lagged concentrations and indicate the frequency of statistically significant positive associations. The results are presented in colour-gradient matrices with the number of associations for every combination of patient strata, pollutant, and lag in corresponding cells. The highest number of the associations was 8 (of 12 possible) obtained for the same day exposure to carbon monoxide, nitrogen dioxide, and days with elevated air quality health index (AQHI) values. For carbon monoxide, the number of the associations decreases with the increasing lags. For this air pollutant, there were almost no associations after 8 days of lag. In the case of nitrogen dioxide, the associations persist even for longer lags. The numerical values obtained from the models are provided for every pollutant. The constructed matrices are a useful tool to analyze the impact of ambient air pollution concentrations on public health.

## Introduction

There is a rich literary body on the relationship between short-term changes in air pollution concentration levels and health problems. The majority of the conducted and published studies are focused on respiratory health problems and their various categories, such as acute respiratory tract infections, asthma, and chronic obstructive pulmonary disease (Grzywa-Celinska et al. 2020; Szyszkowicz and Kousha 2014). There are likewise many studies related to cardiac conditions; however in general, these investigations focus on specific cardiac pathologies (Stieb et al. 2009). In recent years, the association between other health problems and elevated concentrations of ambient air pollution has been increasingly recognized (Braithwaite et al. 2019; Schraufnagel et al. 2019; Szyszkowicz and Rowe 2016). However, these works also focused on specific conditions such as depression, stroke, or skin disease, or focused on a specific air pollutant. Consequently, there is a lack of studies featuring an all-encompassing approach where the health impacts of the most common air pollutants are studied on an array of strata.

The most customary relationships studied between air pollution and a group of health conditions are revealed by a simple search using the PubMed database. When the keywords used are “air pollution respiratory”—12,131 papers are returned, “air pollution cardiac”—3413, “air pollution skin”—1567, and “air pollution depression”—490 papers, indicating that the interaction of air pollution with the respiratory system is most frequently studied. However, public awareness of other effects of air pollutants is increasing. For example, The Guardian (2020) published an article which provides a visual representation of the results presented in the work (Schraufnagel et al. 2019).

In this study, the associations between the number of emergency department (ED) visits in Toronto, Canada, and ambient urban air pollution concentration levels were studied. The project was based on a previous study (Szyszkowicz 2019a), in which the relationship between the air quality health index (AQHI, an indicator composed of three air pollutants) and the number of ED visits for all health problems was studied. In the aforementioned study, 1,199,926 ED visits were identified, belonging to all 18 chapters of health problems specified by the International Classification of Diseases 9th Revision (ICD-9) codes in Edmonton, Canada (from April 18, 1998, to March 31, 2002). The analysis was performed by sex and for lagged exposures, and the associations were found to be positive and statistically significant. The concentration-response curves in the form of non-linear parametric functions were also estimated (Szyszkowicz 2018). At least two other studies investigating the broad associations between air pollution and health emergencies were previously performed (Phung et al. 2018; Wei et al. 2019), where the impact of only fine particulate matter (PM_2.5_) on all ICD-9 chapters was considered. PM_2.5_ (lag 0–2) linked to a wide variety of hospital admissions in US Medicare population.

To provide a more complete picture of the impact of air pollution as a continuation of the previously mentioned publications, this work considers the effect of many pollutants on 12 various types of diseases grouped by the ICD-10 chapters. The diseases in one common chapter, for example “Diseases of the respiratory system”, are analyzed collectively as one health problem without distinguishing between them. The estimated associations with air pollution are thus obtained for 12 various disease chapters. This presentation visualizes the compounded risk of certain air pollutants on many disease groups for specific combinations of age, sex, season, and lag (0–14 days), thus allowing the identification of particularly at-risk strata.

## Methods

The location of this study was Toronto, Canada. The area of the study is well determined by the census division (CD). In the year 2016, the enumerated population of the Toronto CD was 2,731,571 people. Health data and environmental data (ambient air pollutant concentration levels, temperature, and relative humidity) were restricted to the area determined by the CD coordinates. The period of the study was limited and defined by the available data resulting in a period from April 2004 to December 2015.

The health records were drawn from the National Ambulatory Care Reporting System database (NACRS 2020), a health reporting system for ED cases in Canada. The database represents more than 97% of the ED visits in the province of Ontario, where the city of Toronto is located. ED visits for different groups of diseases were identified by applying the International Classification of Diseases 10th Revision (ICD-10) codes. Twelve chapters of diseases categorized in the ICD-10 classification system were extracted. The chapters were chosen on the basis of previously known and reported associations of these health conditions with ambient air pollution (Braithwaite et al. 2019; Szyszkowicz and Rowe 2016; Szyszkowicz 2019a; Szyszkowicz et al. 2020). The following diseases, grouped by chapter, were analyzed and identified by the ICD-10 codes: A00–B99, Certain infectious and parasitic diseases; F00–F99, Mental and behavioural disorders; G00–G99, Diseases of the nervous system; H00–H59, Diseases of the eye and adnexa; H60–H95, Diseases of the ear and mastoid process; I00–I99, Diseases of the circulatory system; J00–J99, Diseases of the respiratory system; K00–K93, Diseases of the digestive system; L00–L99, Diseases of the skin and subcutaneous tissue; M00–M99, Diseases of the musculoskeletal system and connective tissue; N00–N99, Diseases of the genitourinary system; and S00–T98, Injury, poisoning and certain other consequences of external causes.

The National Air Pollution Surveillance (NAPS) Program (managed by Environmental and Climate Change Canada) Canada-Wide Air Quality Database (CWAQD) was used to extract data on six ambient air pollutant concentrations: carbon monoxide (CO), nitrogen dioxide (NO_2_), ozone (O_3_), daily maximum 8-h ozone (O_3_H8), and fine particulate matter (PM_2.5_), where the particles are not greater than 2.5 μm in diameter (NAPS 2020). These environmental data, air pollution, and weather were already used in another multi-country study (Vicedo-Cabrera et al. 2020)

In addition, the air quality health index (AQHI) was calculated, using its three constituent air pollutants: NO_2_, O_3_, and PM_2.5_. The coefficients used in the formula are based on risk of mortality in large Canadian cities (Stieb et al. 2008). Another index, designated here as AQHIX, was also calculated. In the calculation, the concentration of O_3_ is replaced with the concentration of O_3_H8. As the AQHI index is an amalgamate of three air pollutants, this index construction allows emphasis of the presence of ozone in multi-pollutant exposures.

Statistical models based on the case-crossover (CC) method (Maclure 1991) were constructed and the time-stratified approach to identify control periods for cases (Janes et al. 2005) was used. In the standard CC method, conditional logistic regression is typically applied to health events. Here, events were grouped and represented as their daily counts. In this case, conditional Poisson regression models were created for daily counts (Armstrong et al. 2014; Szyszkowicz 2006, 2019b, Szyszkowicz 2020). The counts were grouped by applying the calendar hierarchical clusters in the form “year: month: day of week”. In these statistical models, temperature and relative humidity are represented in the form of natural splines. Air pollution concentrations and weather factors were lagged by the same number of days, from 0 to 14.

For each group of the diseases (an ICD-10 chapter) and for each considered air pollutant (eight analyzed including multi-pollutant indexes), a matrix composed of values of 0 and 1 was constructed. The matrix had 18 rows and 15 columns. The rows correspond to various 18 strata subgroups (sex, age group, season) of the persons registered and diagnosed at ED for the considered diseases. The columns correspond to the specific lag (0–14 days). Values of the estimated relative risks were not presented; instead, qualitative results of the study are generated. Value 1 was assigned to a cell in case of a positive statistically significant association for a particular disease group strata-air pollutant/lag combination; otherwise, a value of 0 was assigned (for both negative and insignificant associations). A *p* value < 0.05 was assumed statistically significant.

Finally, all 12 matrices (constructed for each ICD-10 chapter) were summed by values in corresponding cells. Eight matrices (by air pollutants) were obtained with possible values ranging from 0 to 12. These matrices can be interpreted as an impact of air pollutant on the number of ED visits.

## Results

Tables 1 and 2 list the frequencies of ED visits for the 12 considered health conditions identified by the ICD-10 code ranges. Full names of the ICD-10 code chapters are provided in the table notes. The 18 strata classify the patients by sex (all, female, male), age group (in age ranges: [0–10], [11–60], [61, 61+]), and two seasons (warm: April–September, cold: October–March). Given the large scope of statistical analysis in this study, it may be of importance to determine the number of associations using a lower *p* value. The analysis was run for a *p* value of 0.001 (b), compared to the number of associations for a *p* value of 0.05 (a). The lowest percentages (calculated by the formula % = *b*/a*100%) of persistent associations were 5.6% for diseases of the skin, injury (7.8%), and infectious diseases (9.6%). The highest persistence of associations was for diseases of the eye (33.6%), diseases of the genitourinary system (27.8%), and diseases of the digestive system (26.8%).

**Table 1:** The frequency of ED visits for some diseases. Toronto, Canada, April 2004–December 2015

Strata/ICD-10	A00-B99	F00-F99	G00-G99	H00-H59	H60-95	I00-I99
All	339,644	450,770	140,511	429,629	188,997	484,967
Female	177,619	214,485	83,602	244,710	94,522	235,301
Male	162,025	236,285	56,909	184,919	94,475	249,666
Warm all	161,578	235,302	72,880	225,426	94,368	246,331
Warm female	84,446	111,235	43,432	128,272	47,134	119,641
Warm male	77,132	124,067	29,448	97,154	47,234	126,690
Cold all	178,066	215,468	67,631	204,203	94,629	238,636
Cold female	93,173	103,250	40,170	116,438	47,388	115,660
Cold male	84,893	112,218	27,461	87,765	47,241	122,976
Age 0–10 all	95,377	2587	4292	17,280	59,956	1764
Age 0–10 female	42,933	906	1978	7845	25,738	1286
Age 0–10 male	52,444	1,681	2,,314	9,435	34,218	1990
Age 11–60 all	185,907	383,299	93,633	143,630	101,682	169,020
Age 11–60 female	100,324	177,809	57,456	75,923	52,946	73,271
Age 11–60 male	85,583	205,490	36,177	67,707	48,736	95,749
Age 60+ all	58,360	64,884	42,586	268,719	27,359	312,671
Age 60+ female	34,362	35,770	24,168	160,942	15,838	160,744
Age 60+ male	23,998	29,114	18,418	107,777	11,521	151,927

A00–B99, Certain infectious and parasitic diseases; F00–F99, Mental and behavioural disorders; G00–G99, Diseases of the nervous system; H00–H59, Diseases of the eye and adnexa; H60–H95, Diseases of the ear and mastoid process; I00–I99, Diseases of the circulatory system

**Table 2:** The frequency of ED visits for some diseases. Toronto, Canada, April 2004–December 2015

Strata/ICD-10	J00-J99	K00-K99	L00-L99	M00-M99	N00-N99	S00-T98
All	852,624	993,364	342,939	691,703	882,918	2,298,581
Female	421,903	514,336	167,574	368,089	531,876	1,044,560
Male	430,721	479,028	175,365	323,614	351,042	1,254,021
Warm all	376,931	507,627	192,955	366,557	460,044	1,272,513
Warm female	184,694	263,423	94,282	195,811	277,806	569,346
Warm male	192,237	244,204	98,673	170,746	182,238	703,167
Cold all	475,693	485,737	149,984	325,146	422,874	1,026,068
Cold female	237,209	250,913	73,292	172,278	254,070	475,214
Cold male	238,484	234,824	76,692	152,868	168,804	550,854
Age 0–10 all	222,107	66,407	28,647	15,954	28,579	276,088
Age 0–10 female	91,080	28,915	13,162	7023	12,901	116,404
Age 0–10 male	131,027	37,492	15,485	8931	15,678	159,684
Age 11–60 all	434,200	577,811	227,861	465,555	547,864	1,578,158
Age 11–60 female	227,051	298,664	108,173	233,025	384,360	660,210
Age 11–60 male	207,149	279,147	119,688	232,530	163,504	917,948
Age 60+ all	196,317	349,146	86,431	210,194	306,475	444,335
Age 60+ female	103,772	186,757	46,239	128,041	134,615	267,946
Age 60+ male	92,545	162,389	40,192	82,153	171,860	176,389

J00–J99, Diseases of the respiratory system; K00–K93, Diseases of the digestive system; L00–L99, Diseases of the skin and subcutaneous tissue; M00–M99, Diseases of the musculoskeletal system and connective tissue; N00–N99, Diseases of the genitourinary system; S00–T98, Injury, poisoning and certain other consequences of external causes

**Fig. 1:**
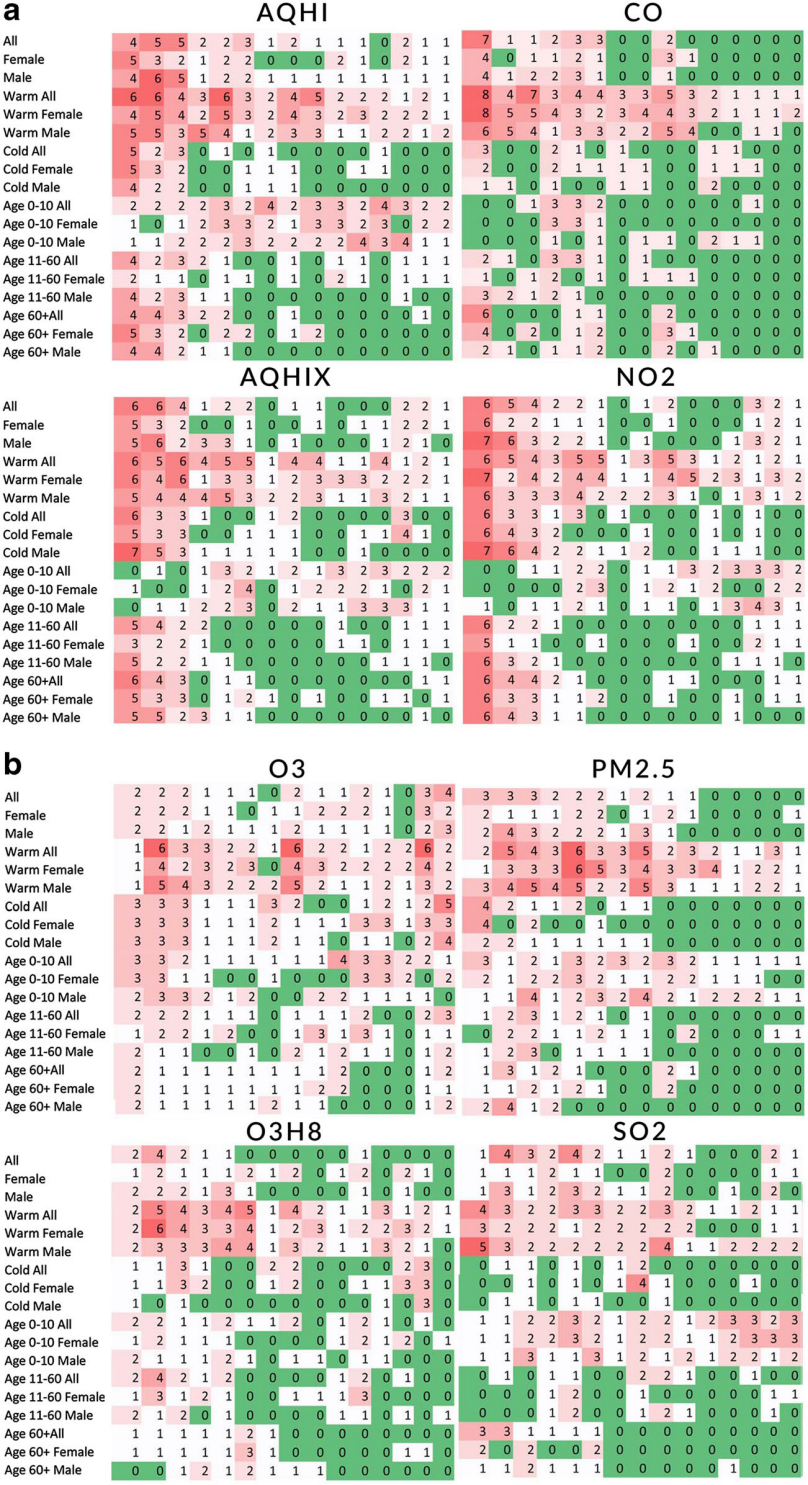
**a** Frequencies of the positive associations for AQHI, AQHIX, CO, and NO_2_. The columns indicate lags from 0 to 14. **b** Frequencies of the positive associations for O_3_, O_3_H8, PM_2.5_, and SO_2_. The columns indicate lags from 0 to 14

Figure 1 a and b show matrices containing the association marker sums for all 8 air pollutants. The colour range from green to red is used to discriminate values but is not linearly scaled by value. A darker red indicates more positive associations, while dark green indicates no positive associations for any ICD-10 code chapter. The maximum expected sum in a cell is 12 (positive associations for that cell in each disease chapter); however, the maximum observed here is 8. The corresponding values of numerical estimations of slope and associated standard error can be found at https://github.com/szyszkowiczm/ResultsToronto. These values allow calculating relative risks and their 95% confidence intervals for an increase in air pollution concentration levels.

Using the constructed matrices, the air pollutant with the highest number of the associations was identified. In alphabetical order of the air pollutant names (AQHI–SO_2_), there were 441, 432, 294, 427, 427, 300, 354, and 292 positive associations, respectively. Notably, AQHI has the highest association count among the pollutant factors studied. The highest number of associations for an individual stratum is 50 for “all patients” in the warm season for CO concentrations. Table 3 summarizes the results for the all 18 considered strata. In the bottom row, the table provides the maximum number of associations for the considered pollutant. In the last row, the percentage of positive statistically significant results among all tested is presented.

**Table 3:** The numbers of positive associations summarized for 15 lags by 8 air pollutants. Toronto, Canada, April 2004–December 2015

Strata/pollutant	AQHI	AQHIX	CO	NO_2_	O_3_	O_3_H8	PM_2.5_	SO_2_
All	31	28	19	29	23	11	20	24
Female	22	19	13	18	22	17	14	11
Male	29	25	14	29	21	13	20	19
Warm all	49	50	50	47	41	39	44	32
Warm female	44	42	48	45	36	39	44	22
Warm male	40	41	37	36	36	33	41	34
Cold all	13	19	9	19	28	15	12	6
Cold female	15	21	11	18	30	18	7	9
Cold male	11	21	7	28	24	6	10	5
Age 0–10 all	38	25	10	24	29	17	27	30
Age 0–10 female	28	19	7	15	19	13	21	29
Age 0–10 male	32	24	8	20	21	12	29	24
Age 11–60 all	18	17	11	14	19	15	11	9
Age 11–60 female	13	13	8	13	19	14	14	6
Age 11–60 male	12	14	9	15	15	10	10	8
Age 60+ all	17	17	10	21	15	8	11	10
Age 60+ female	17	19	13	20	15	11	10	6
Age 60+ male	12	18	10	16	14	9	9	8
Maximum	49	50	50	47	41	39	44	34
% of all tested	35.0	34.3	23.3	33.9	33.9	23.8	28.1	23.2

## Discussion

This study explored associations between elevated levels of ambient air pollution in Toronto, Canada, and a large spectrum of health conditions. This broad approach to statistical associations between air pollution levels and ED visits sheds more light on how considerable the impact of air quality is on the overall health of Canadian population.

Recently, more studies that examine health conditions other than the traditionally studied respiratory and cardiac pathologies are being performed (Szyszkowicz et al. 2020). In the present study, it was hypothesized that the broad range of health conditions typically not considered by epidemiological studies are likewise associated to air pollution concentration levels. The results obtained here support this assumption, as positive associations for up to 8 ICD-10 chapters were observed for some pollutants, well beyond the typical scope of circulatory and respiratory diseases. This broad range of health effects demonstrates the benefit in pursuing more focused studies that consider the effect of air pollution on specific ICD-10 chapters.

This article proposes an approach to assess the burden of diseases, here measured as the daily number of emergency department visits, in relation to ambient air pollution concentration in the urban area. The daily counts of ED visits were considered to be health outcomes. The counts represent a summation of all visits in the considered chapter of the health classification. Some of these visits are associated (A) with air pollution concentration levels, some are neutral (B), and others may be negatively associated (C). The daily counts composed with (A) and (C) should result with neutral responses—no associations. The combination of (A) and (B) results in (A), and similarly (B) and (C) combined yields (C). In this paper, for each health group, 2160 statistical models were constructed, and despite the low resolution of some ICD chapters (as multiple diseases are grouped under one category), positive associations were observed.

The considered counts of diseases are composites of various types and their reaction to air pollution exposure varies depending on the disease considered. The AQHI was observed to have the highest number of associations, which may be explained by the fact that the index incorporates three air pollutants that may be associated with health conditions individually, or collectively: for example, due to the negative effect of ozone on the respiratory system and nitrogen dioxide on the cardiac system. Our results show that the AQHI is a very effective predictive tool and reveal how strongly NO_2_ is associated with a wide range of ED visit chapters for a short lag time (0–1 days). In the scope of this study, the positive associations between NO_2_ at almost all strata examined and many different health condition chapters show that NO_2_ is a very potent pollutant that can affect a wide range of physiological systems. For lag 0, there were 92 models with positive associations out of a total of 216 models (43%) for NO_2_ among all strata. This could also be a partial explanation as to why the AQHI has the highest total amount of associations. While one would expect ozone to have the highest impact on the AQHI, it seems this is not the case at early lags; rather, it is nitrogen dioxide. This impact is also seen in the AQHIX, where even when emphasizing the effect of ozone, the earliest lags have the highest amount of associations, similar to what was seen for NO_2_.

Some of this study’s limitations involve the interpretation of the findings of this type of observational research. Many statistical models were tested, which may be or not be adequate. The presented patterns of the associations are based on *p* values less than 0.05. Given the limitations including the multi-comparison issue, results should be interpreted with caution and require further examination using data from different locations and different methodologies. The authors acknowledge various possible measurement errors related to disease diagnosis and environmental variables. Another limitation is the assumption that each individual who visits the ED experiences the same exposure to air pollutants. The exposure to air pollution used in this study is an average of concentration levels in Toronto based on seven measurement stations (NAPS 2020), and to limit the error the latter limitation introduces, the stations and EDs are both located in densely urbanized areas.

Finally, many individuals with health complications do not go to the emergency department to seek medical attention. However, this should introduce non-differential misclassification and bias our results towards the null.

In this work, only ambient air pollution was considered. As it is well known, some household and indoor ambient air pollution or mould (Bungau et al. 2019) may also have effect on a human health and by a consequence on ED visits.

In future studies, it would be useful to contrast the results obtained here to other cities using similar methodologies to prove the repeatability of these associations.

## Conclusion

This study assessed the impact of ambient air pollution concentration levels on emergency visits for various disease groups and presented the results by determining the statistical significance of associations between the ED visits and fluctuations in concentrations of pollutants. The results generally indicate that there is a potential increased risk of ED visits for a range of disease groups on the day of or subsequent days post-exposure to elevated ambient air pollution concentration levels. However, the study cannot establish causality given the limitations of epidemiological studies of this nature; however, the authors hope to highlight the broad range of health effects that air pollution can have, of which some relationships have not been deeply investigated.

## Data Availability

The data are presented under the following link: https://github.com/szyszkowiczm/ResultsToronto.
